# Single-Cell Transcriptomics Bioinformatics and Computational Challenges

**DOI:** 10.3389/fgene.2016.00163

**Published:** 2016-09-21

**Authors:** Olivier B. Poirion, Xun Zhu, Travers Ching, Lana Garmire

**Affiliations:** ^1^Epidemiology Program, University of Hawaii Cancer CenterHonolulu, HI, USA; ^2^Molecular Biosciences and Bioengineering Graduate Program, University of Hawaii at ManoaHonolulu, HI, USA

**Keywords:** single-cell genomics, single-cell analysis, bioinformatics, heterogeneity, microevolution

## Abstract

The emerging single-cell RNA-Seq (scRNA-Seq) technology holds the promise to revolutionize our understanding of diseases and associated biological processes at an unprecedented resolution. It opens the door to reveal intercellular heterogeneity and has been employed to a variety of applications, ranging from characterizing cancer cells subpopulations to elucidating tumor resistance mechanisms. Parallel to improving experimental protocols to deal with technological issues, deriving new analytical methods to interpret the complexity in scRNA-Seq data is just as challenging. Here, we review current state-of-the-art bioinformatics tools and methods for scRNA-Seq analysis, as well as addressing some critical analytical challenges that the field faces.

## Introduction

Characterization of genomic signatures in individual patients is a key step toward the realization of precision medicine. Recently, next-generation sequencing (NGS) based RNA expression profiling (RNA-seq) has made broad impacts on biomedical fields. However, population-averaged RNA-seq has limited discovery power, and it can also mask the presence of rare subpopulations of cells (such as cancer stem cells) and thus may overlook important biological insights. The emerging single-cell RNA-Seq (scRNA-Seq) technology is designed to overcome these limitations by investigating expression profiles at the cell level. In just a few years, the number scRNA-Seq experiments has grown beyond exponentially. This new approach offers the potential to revolutionize our understanding of diseases and associated biological processes, with the capacity to reveal the intercellular heterogeneity within a specific tissue at an unprecedented resolution (Yan et al., 2013; Trapnell et al., 2014). Using single-cell level features, we can infer cell lineages (Treutlein et al., 2014), identify subpopulations (Trapnell et al., 2014) and highlight cell-specific biological characteristics (Tang et al., 2010). Moreover, single-cell analyses have already demonstrated their utilities in the clinical applications, ranging from characterizing cancer cells subpopulations (Navin et al., 2011; Patel et al., 2014; Ting et al., 2014), highlighting specific resistance mechanisms (Kim, K. T. et al., 2015; Miyamoto et al., 2015) to being used as diagnostic tools (Ramsköld et al., 2012; Kvastad et al., 2015).

**Figure 1:**
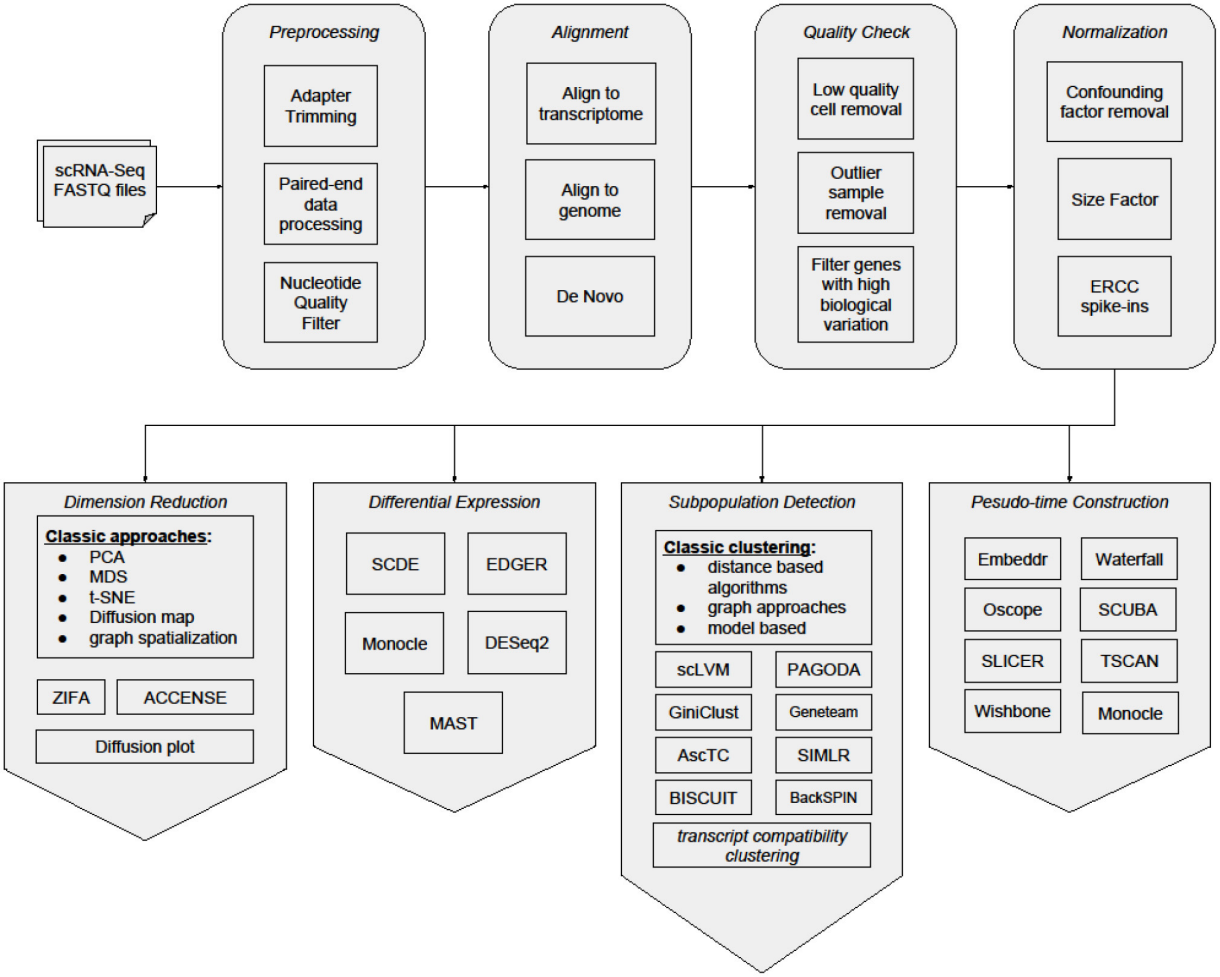
**General workflow of Single-cell analysis**.

Despite the expansion of scRNA-Seq studies and rapid maturing of experimental methods, major analytical challenges remain as the consequences of experimentation. One major challenge is that scRNA-Seq datasets present a very high level of noise (Brennecke et al., 2013; Kharchenko et al., 2014). Much of the noise is due to the nature of single-cell technologies. Because of the extremely low amount of starting biological material in the single cell, amplification processes are required. These procedures are prone to distortion and contamination (Leng et al., 2015). To tackle these issues, rigorous efforts have been made to develop analytical methods for scRNA-Seq data. Here, we summarize current state-of-the-art bioinformatics analysis tools and methods for scRNA-Seq (Figure 1 and Table 1), and address some critical analytical challenges that we are facing. The first section describes specific pre-processing steps for noise removal of scRNA-Seq datasets. The second section reviews specific scRNA-Seq bioinformatics analysis procedures with emphasis on subpopulation detection. The third section focuses on microevolution analysis for scRNA-Seq data. In the last section, we highlight the challenges to be addressed and work to be accomplished in scRNA-Seq bioinformatics field.

**Table 1:** **List of single-cell analytical tools mentioned in this chapter**.

**Category**	**Tool name**	**References**	**Availability**
Preprocessing	cutadapt	Martin, 2011	https://cutadapt.readthedocs.org/en/stable/index.html
Preprocessing	Trimmomatic	Bolger et al., 2014	http://www.usadellab.org/cms/?page=trimmomatic
Preprocessing	FASTQC	Andrews, 2010	http://www.bioinformatics.babraham.ac.uk/projects/fastqc/
Preprocessing	SolexaQA	Cox et al., 2010	http://solexaqa.sourceforge.net/
Preprocessing	BIGpre	Zhang et al., 2011	https://sourceforge.net/projects/bigpre/
Preprocessing	HTQC	Yang et al., 2013	https://sourceforge.net/projects/htqc/
Preprocessing	SinQC	Jiang, P. et al., 2016	http://www.morgridge.net/SinQC.html
Preprocessing	SCell	Diaz et al., 2016	https://github.com/diazlab/scell
Preprocessing	celloline	Ilicic et al., 2016	https://github.com/Teichlab/celloline
Alignment	Tophat	Trapnell et al., 2009; Kim et al., 2013	https://ccb.jhu.edu/software/tophat/index.shtml
Alignment	RSEM	Li and Dewey, 2011	http://deweylab.github.io/RSEM/
Alignment	GSNAP	Wu et al., 2016	http://research-pub.gene.com/gmap/
Alignment	STAR	Dobin and Gingeras, 2015	https://github.com/alexdobin/STAR
Alignment	Mapsplice	Wang et al., 2010	http://www.netlab.uky.edu/p/bioinfo/MapSplice2
Quantification	Cufflinks	Trapnell et al., 2010	http://cole-trapnell-lab.github.io/cufflinks/
Quantification	HISAT	Kim, D. et al., 2015	https://ccb.jhu.edu/software/hisat2/index.shtml
Quantification	HTSeq	Anders et al., 2014	http://www-huber.embl.de/HTSeq/doc/overview.html
Quantification	FeatureCounts	Liao et al., 2013	http://bioinf.wehi.edu.au/featureCounts/
Quantification	Kallisto	Bray et al., 2016	https://pachterlab.github.io/kallisto/about.html
Gene filtering	OEFinder	Leng et al., 2016	https://github.com/lengning/OEFinder
Cofounding factor removal	scLVM	Buettner et al., 2015	https://github.com/PMBio/scLVM
Cofounding factor removal	COMBAT	Johnson et al., 2007	https://github.com/brentp/combat.py
Normalization	GRM	Ding et al., 2015	http://wanglab.ucsd.edu/star/GRM/
Normalization	BASICS	Vallejos et al., 2015	http://journals.plos.org/ploscompbiol/article/asset?unique&id=info:doi/10.1371/journal.pcbi.1004333.s009
Normalization	SAMstrt	Katayama et al., 2013	https://github.com/shka/R-SAMstrt
Normalization	Deconvolution	Aaron et al., 2016	https://github.com/MarioniLab/Deconvolution2016
Dimension Reduction	pcaReduce	Zurauskiene and Yau, 2015	https://github.com/JustinaZ/pcaReduce
Dimension Reduction	*t*-SNE	der Maaten and Hinton, 2008	https://lvdmaaten.github.io/tsne/
Dimension Reduction	ACCENSE	Shekhar et al., 2014	http://www.cellaccense.com/
Dimension Reduction	ZIFA	Pierson and Yau, 2015	https://github.com/epierson9/ZIFA
Differential Expression	SCDE	Kharchenko et al., 2014	http://hms-dbmi.github.io/scde/
Differential Expression	PAGODA	Fan et al., 2016	http://hms-dbmi.github.io/scde/
Differential Expression	EdgeR	Robinson et al., 2010	https://bioconductor.org/packages/release/bioc/html/edgeR.html
Differential Expression	DESeq2	Love et al., 2014	https://bioconductor.org/packages/release/bioc/html/DESeq2.html
Differential Expression	MAST	Finak et al., 2015	https://github.com/RGLab/MAST
Subpopulation Detection	GiniClust	Jiang, L. et al., 2016	https://github.com/lanjiangboston/GiniClust
Subpopulation Detection	Geneteam	Harris et al., 2015	
Subpopulation Detection	AscTC	Ntranos et al., 2016	https://github.com/govinda-kamath/clustering_on_transcript_compatibility_counts
Subpopulation Detection	SIMLR	Wang et al., 2016	https://github.com/BatzoglouLabSU/SIMLR
Subpopulation Detection	BISCUIT	Prabhakaran et al., 2016	http://www.c2b2.columbia.edu/danapeerlab/html/pub/prabhakaran16-supp.pdf
Subpopulation Detection	BackSPIN	Zeisel et al., 2015	https://github.com/linnarsson-lab/BackSPIN
Microevolution	Moncole	Trapnell et al., 2014	http://cole-trapnell-lab.github.io/monocle-release/
Microevolution	embeddr	Campbell et al., 2015	https://github.com/kieranrcampbell/embeddr
Microevolution	SCUBA	Marco et al., 2014	https://github.com/gcyuan/SCUBA
Microevolution	Oscope	Leng et al., 2015	https://www.biostat.wisc.edu/~kendzior/OSCOPE/
Microevolution	SLICER	Welch et al., 2016	https://github.com/jw156605/SLICER
Microevolution	TSCAN	Ji and Ji, 2016	http://bioconductor.org/packages/release/bioc/html/TSCAN.html
Workflow	SINCERA	Guo et al., 2015	https://research.cchmc.org/pbge/sincera.html

Links for their availability are attached.

## Data preprocessing and noise removal

### Quality control

scRNA-Seq experiments generate FASTQ files from the sequencing machine, which contain millions of reads composed of RNA sequences and add-on sequences (UMI tag and the cell tag etc). These reads need to be pre-processed before being aligned back to the reference genome. For scRNA-seq, pre-processing and quality control (QC) analyses similar to bulk RNA-seq are used. Cutadapt (Martin, 2011) is a tool that removes adapter sequences, and Trimmomatic (Bolger et al., 2014) performs quality-based trimming in addition to removing adapter sequence. These tools are commonly used in scRNA-seq experiments (Treutlein et al., 2014; Handel et al., 2016; Hou et al., 2016). Other generic quality control tools such as FASTQC or HTQC (Yang et al., 2013) might also be useful to produce quality metrics. Finally, it is worth noting that platform-specific QC tools such as SolexaQA (Cox et al., 2010) provide QC pipelines specific for Illumina sequencing, with trimming and quality-based filtering.

Other QC procedures for scRNA-seq involve the analysis of the expression of housekeeping genes (Ting et al., 2014; Treutlein et al., 2014), overall gene expression patterns (Zeisel et al., 2015) and the number of genes or reads detected per cell (Kumar et al., 2014). However, one issue of these approaches is that the thresholds chosen for filtering are arbitrary and should differ according to the dataset (Jiang, P. et al., 2016). SinQC (Jiang, P. et al., 2016) and SCell (Diaz et al., 2016) are two QC tools specifically designed for scRNA-seq data. SinQC uses sequencing library quality to confirm gene expression outliers. It computes different quality metrics (e.g., total number of mapped reads, mapping rate and library complexity) to identify a user-specified fraction of the dataset as noise. SCell is a versatile tool that allows for outlier detection. It estimates genes that are expressed at the background level using Gini index, which measures statistical dispersion, and removes samples whose background fraction is significantly higher than the average. Recently, a new mapping and quality assessment pipeline Celloline detects low quality cells from expression profiles, using curated biological and technical features (Ilicic et al., 2016).

### Alignment

To our knowledge, there are currently no specific aligners dedicated to scRNA-seq, and scRNA-seq studies use existing aligners made for bulk RNA-Seq. Tophat is one of the most popular aligners capable of detecting novel splice (Trapnell et al., 2009; Kim et al., 2013), and it is widely used in scRNA-seq studies (Treutlein et al., 2014; Fan et al., 2016; Freeman et al., 2016; Handel et al., 2016; Hou et al., 2016). RNA-Seq by Expectation Maximization, or RSEM, is a popular framework that includes an aligner (Li and Dewey, 2011). It is also used in some scRNA-seq studies (Gao et al., 2016; Kimmerling et al., 2016; Meyer et al., 2016). Other aligners used in scRNA-Seq studies include MapSplice (Wang et al., 2010), GSNAP (Brennecke et al., 2013; Buettner et al., 2015; Wu et al., 2016), and STAR (Dobin and Gingeras, 2015; Moignard et al., 2015; Petropoulos et al., 2016). Among these aligners, TopHat and STAR were found to be about one to two magnitudes faster than GSNAP and MapSplice (Engström et al., 2013). More recently developed aligners include Kallisto (Bray et al., 2016) and HISAT (Kim, D. et al., 2015). Kallisto uses pseudo-alignment with hashing de Bruijn graphs and avoids alignment altogether, which drastically improves the speed of expression quantification. HISAT (hierarchical indexing for spliced alignment of transcripts) seems also promising in term of the speed and accuracy. It is worth mentioning that some major scRNA-Seq methods do not get enough coverage across the gene to measure alternative splicing, therefore algorithms for isoform measurements are not as critical in scRNA-Seq, at least at this stage.

### Feature quantification

Feature quantification is the process of converting alignment results into a gene expression profile. An expression profile is conventionally represented as a numeric matrix where rows are genes and columns are cells. Each entry in the matrix is the abundance of a particular gene or transcript in a particular sample. Just as is the case for aligners, most scRNA-Seq studies use canonical feature quantification methods applied to bulk RNA-Seq.

Quantification methods for gene expression differ dramatically. The simplest approach, employed by programs such as HTSeq (Anders et al., 2014) and FeatureCounts (Liao et al., 2013), is to count the number of reads located within the boundaries of a gene (Liao et al., 2013; Anders et al., 2014). These programs have simple but flexible parameters for determining read counts in the case of overlapping genes, and were used in some scRNA-Seq studies (Brennecke et al., 2013; Moignard et al., 2015; Fan et al., 2016; Handel et al., 2016). More sophisticated approaches calculate probabilistic estimates of gene expression. For example, RSEM and Cufflinks both employ a maximum likelihood approach (Trapnell et al., 2010; Li and Dewey, 2011). These programs are based on statistical models where reads in a RNA-Seq sample are observed random variables predicted from the latent variables, such as the transcript sequence, strand and length. The new Kallisto pipeline (Bray et al., 2016) as described before, is shown to have up to two orders of magnitude speed improvement over previous aligner-quantifier combinations (Ntranos et al., 2016). Interestingly, while probabilistic approaches are conceptually more refined, simple counting programs such as HTSeq and FeatureCounts showed comparable or even stronger performance (Chandramohan et al., 2013; Fonseca et al., 2014), suggesting that these probabilistic models are yet to be improved.

Given the uncertainties of quantifying fragments post-amplification, a new technique was shown to reduce amplification noise by introducing random sequences called unique molecular identifiers, or UMIs (Islam et al., 2014). UMIs are tagged on individual RNA molecules before amplification and used for tracking transcripts directly rather than using sophisticated statistical modeling. This approach may lead to a different workflow than conventional fragment-based quantification methods (e.g., gene filtering and normalization).

### Gene filtering

Due to the high level of noise in scRNA-Seq datasets, it is necessary to filter out low quality genes and samples. Various practices have been made to filter out genes that are expressed in too few samples (Brennecke et al., 2013; Treutlein et al., 2014; Petropoulos et al., 2016). Usually, a gene is defined as “expressed” by a minimal expression level threshold. For experiments that quantify gene expression with fragment counting, an FPKM (Fragment per Kilobase per Million Reads) threshold is appropriate. Common FPKM thresholds are 1 (Freeman et al., 2016) and 10 (Petropoulos et al., 2016). Other studies also set the threshold by Transcript Per Million (TPM) instead of FPKM (Meyer et al., 2016). Yet better filtering reference could come from External RNA Controls Consortium (ERCC) spike-ins added to the experiment, which provides calibration of the relative amount of starting material (Brennecke et al., 2013; Treutlein et al., 2014).

Recently, specific methods have been developed to filter genes from scRNA-seq dataset. OEFinder is designed to identify artifact genes from scRNA-seq experiments using the Fluidigm C1 platform for cell capture (Leng et al., 2016). For experiments that quantify gene expression with UMI counting, one can directly set up a molecule number threshold, e.g., 25 (Zeisel et al., 2015). It is also recommended to remove UMIs that have reads <1/100 of average non-zero UMI reads, in order to avoid erroneous UMIs generated during amplification.

### Removal of confounding factors

When the entire data set consists of several runs of experiments with potentially varied conditions, systematic variations called batch effects might be introduced. These artifacts may pose substantial problems to downstream statistical analysis, or even mask biological signals. For studies concerning over-dispersion of gene expression, it is necessary to factor out the extra variance caused by the systematic differences between batches (Fan et al., 2016). The appropriate way to compensate for batch effect depends on the quantification method as well as the downstream analysis. For most studies batch effects can be eliminated by using down-sampling methods, however the complexity is reduced (Wang et al., 2012; Dey et al., 2015; Grün and van Oudenaarden, 2015). For studies that use traditional fragment counting, COMBAT (Johnson et al., 2007) is a batch effect eliminating method based on empirical Bayes frameworks and purports to be robust to outliers for small sample sizes. It was originally designed for microarray data but was used in scRNA-Seq experiments (Kim, K. T. et al., 2015). Although unsupervised batch effect detection or removal methods exist (Leek, 2014), the batches called by such methods often correlate highly with subpopulations detected by other scRNA-Seq methods (Finak et al., 2015). Since it is usually desirable to consider subpopulations for valuable biological insights, unsupervised batch effect removal methods should be used with discretion in single-cell experiments.

Besides batch-effect removal, it is also important to remove technical variability within the noise. The technical noise level of a genes correlates with its average expression level. Thus, a probabilistic model can be built to fit this correlation using technical spike-ins and further infer the biological variability of each gene (Brennecke et al., 2013). For most studies, it is also desirable to avoid the ubiquitous cell-cycle induced variation to mask other interesting biological variations. scLVM is a package that tries to introduce a cell-cycle factor removal step before subpopulations detection (Buettner et al., 2015). Recently, a new package called ccRemover was developed to remove the principal components that are identified as cell-cycle affected, which claimed to perform better than scLVM in several simulated and real datasets (Barron and Li, 2016).

### Normalization

In scRNA-seq experiments, technical factors such as read depth, cell capture efficiency, 3′ bias or full sequence coverage due to particular library prep methods, might differ among different scRNA-Seq data sets. Thus, raw read counts should be normalized before downstream analyses. This procedure maximally ensures that the difference between the values in the matrix correctly reflects the abundance difference of transcripts or genes between the cells. When experiments are designed with ERCC spike-ins, ERCC can be used as internal controls and serve as anchors for normalization. GRM is a scRNA-seq normalization tool fitting a Gamma Regression Model between the reads (FPKM, RPKM, TPM) and spike-ins (Ding et al., 2015). The trained model is then used to estimate gene expression from the reads. BASICS, another recent workflow, provides a Bayesian model allowing to infer cell-specific normalization factor (Vallejos et al., 2015). This workflow estimates the technical variability using spike-ins. Finally, SAMstrt (Katayama et al., 2013) is an earlier algorithm that applies the resampling normalization procedure of the SAMseq algorithm to spike-ins, which was originally developed for bulk RNA-seq (Li and Tibshirani, 2013).

For experiments without spike-ins, if the quantification is count-based, one can normalize the expression profile by the scaling methods used in DESeq and edgeR etc. (Love et al., 2014). A new specific scRNA-seq procedure proposes a de-convolution approach on the pooled counts of gene expression for multiple cells, thus allows to infer the size factor for individual cells without using spike-ins (Aaron et al., 2016). The authors claimed that their approach improved the accuracy of the normalization compared with existing methods. However, experiments designed with UMIs as mentioned earlier quantify gene expression on an absolute basis and thus they do not need computational normalization.

### Differential expression

Differential expression (DE) analysis is the process of calling gene expression that show statistically significant difference between pre-specified groups of samples. Although DE is typically not the main objective of a single-cell experiment design, as it requires pre-defined grouping information among cells of interest, it is nevertheless common in scRNA-Seq experiments. Simple statistical methods such as *t*-test and Wilcoxon rank sum test are used in scRNA-Seq workflows such as SINCERA (Guo et al., 2015). Interestingly, EdgeR and DESeq2, two DE methods developed for bulk RNA-Seq, gave the best results for some scRNA-Seq data (Schurch et al., 2016).

The dropout event is a unique type of noise of scRNA-Seq that rarely occurs in bulk RNA-Seq experiments. It refers to the phenomenon that a gene is shown expressed abundantly in one cell but not detectable in another cell, as a consequence of the transcript loss in the reverse-transcription step. To account for frequent dropout events and biological variability within cell population, more sophisticated algorithms have been developed for scRNA-Seq data. Single-Cell Differential Expression (SCDE) is a package developed specifically for single-cell differential expression (Kharchenko et al., 2014). The model assumes that observed expression levels in scRNA-Seq data follow a mixture of negative binomial distribution for amplified genes, as proposed before (Anders and Huber, 2010); and a low-mean poisson distribution for dropout genes, as is observed in transcriptionally silenced genes. This model is then fit using Expectation Maximization (EM) algorithm (Kharchenko et al., 2014). It claimed higher sensitivity of differentially expressed genes compared to DESeq and CuffDiff. More recently, PAGODA improved upon SCDE's method in several aspects, including optimization of the computational process and a refined model for better fitting (Fan et al., 2016). MAST is another scRNA-Seq differential expression detection method that uses a two-part generalized linear model and adjusts for the fraction of cells that express a certain gene (Finak et al., 2015).

Another challenge unique to scRNA-Seq is that some genes may exhibit bimodality, meaning that the expression levels across a group of cells concentrate around two modes instead of one. A beta-Poisson distribution was proposed in order to provide a more accurate differential expression analysis that captures bimodality (Vu et al., 2016). Another tool Monocle (Trapnell et al., 2014) also has a module for differential expression, which fits the data with a non-parametric generalized additive model. Finally, the workflow of BASICS as described earlier, provides an criterion to detect high- or low-variable genes within the single cells dataset (Vallejos et al., 2015). However, it is not clear which methods have generally superior performance.

## Subpopulation and module detection

### General machine-learning approaches

Different classical unsupervised approaches have been used to highlight single cell subgroups among a population. Principal Component Analysis (PCA) and its variants (e.g., Robust PCA and Kernel PCA) have been used in different single cell studies (Amir et al., 2013; Yan et al., 2013; Pollen et al., 2014; Trapnell et al., 2014; Treutlein et al., 2014; Satija et al., 2015; Fan et al., 2016; Ilicic et al., 2016). *K*-means and other distance based clustering algorithms such as hierarchical clustering or WARD are also widely used (Yan et al., 2013; Jaitin et al., 2014; Kharchenko et al., 2014; Lohr et al., 2014; Marco et al., 2014; Pollen et al., 2014; Shin et al., 2015). For example, Jaitin et al. combined hierarchical clustering and probabilistic mixture models to classify single cells from different tissues (Jaitin et al., 2014). A refined clustering method called pcaReduce (Zurauskiene and Yau, 2015) was designed for scRNA-Seq. It iteratively uses PCA combined with *K*-means to produce the hierarchical tree of the cells. For distance metrics employed by these methods, Euclidean distance, Pearson and Spearman correlation coefficients have been popular (though may not be optimal) choices (Pollen et al., 2014; Rotem et al., 2015).

### Machine-learning approaches tailored for scRNA-Seq analysis

More sophisticated machine-learning algorithms have great potentials to overcome some issues of scRNA-Seq functional analysis. A main issue of scRNA-Seq analysis is that gene expression data cannot be expressed as a linear combination of the relationships between two cells in general (Buettner and Theis, 2012; Bendall et al., 2014; Levine et al., 2015). Also classical similarities (such as cosine or Euclidean distances) are less meaningful as the dimensionality increases (Beyer et al., 1999), and may not be appropriate for scRNA-Seq (Xu and Su, 2015). Possible irrelevant associations may arise with inappropriate metrics, while searching for the nearest neighbors on noisy data (Balasubramanian and Schwartz, 2002). Adequate analytical methods for scRNA-Seq data should also be able to highlight “rare events,” such as the small fraction of metastatic cancer cells amongst a large cell population (Bose et al., 2015; Shin et al., 2015). We describe the scRNA-Seq specific algorithms below in the order of dimension reduction, clustering, and other clustering variant methods. The datasets that were used to test these algorithms are listed in Table 2.

**Table 2:** **Description of the main datasets for subpopulation and module detection analysis**.

**Dataset description**	**Accession**	**References**	**Species**	**Number of cells**	**Original analysis**	**Applied algorithms**
Cortex and hippocampus cells	GSE60361	Zeisel et al., 2015	Mouse	3005	BackSPIN	Geneteam, PAGODA, AscTC, BISCUIT, GiniClust
11 different cell types	SRP041736	Pollen et al., 2014	Human	301	PCA and hierarchical clustering	ZIFA, SILMR, pcaReduce
Myoblast differentiation	GSE52529	Trapnell et al., 2014	Human	372	MONOCLE	ZIFA, AscTC, TSCAN, Embeddr
Embryomic T-cells under different cell cycle stages	E-MTAB-2512	Buettner et al., 2015	Mouse	182	scLVM	ZIFA, SLIMR
Preimplementation embryos and embryonic stem cells at different stages	GSE36552	Yan et al., 2013	Human	124	PCA and hierarchical clustering	scLVM, SNN-Cliq
Cells from developing bronchioalveolar at four different stages of development	GSE52583	Treutlein et al., 2014	Mouse	202	PCA and hierarchical clustering	SLICER, EMBEDDR

Among the dimension reduction methods, Zero-inflated factor analysis (ZIFA) algorithm is a new method that includes dropout events by representing the probability of gene dropout as an exponential function of its mean expression (Pierson and Yau, 2015). Using a latent variable model based on factor analysis, ZIFA reduces the dimension of scRNA-Seq dataset and allows the probability of each gene expression to be zero. Experiments in the original study suggest that ZIFA is a more robust alternative to PCA. As mentioned earlier, scLVM is another method for identifying cell subpopulations, which features removal of confounding factor like cell-cycle effects (Buettner et al., 2015). It first computes cell-to-cell covariance using a set of marker genes related to biological hidden factors of interest (such as the cell cycle). Another approach, PAGODA as mentioned before, uses a weighted PCA to characterize multiple aspects of heterogeneity in mouse neuronal progenitors (Fan et al., 2016). PAGODA evaluates over-dispersion of individual genes using error models.

SIMLR is a new clustering method designed to learn a distance metric that best fits the structure of the data. It infers a distance function as a linear combination of several distance metrics (Wang et al., 2016). It is designed to tackle the heterogeneity observed amongst single-cell datasets related to both technological difference across platforms as well as biological difference across studies. In another single-cell clustering approach named analysis of scRNA-seq based on transcript-compatibility counts (AscTC), read counts from scRNA-Seq dataset are transformed into probabilities using transcript-compatibility counts, rather than the conventional transcript abundance (Ntranos et al., 2016). Individual cells are clustered using an affinity propagation algorithm, a derivative of spectral clustering.

A few other hierarchical clustering approaches are worth mentioning. Geneteam is a multi-level recursive clustering method that searches for bipartitions of cells sharing exclusive expression profiles for a subset of genes (Harris et al., 2015). Similarly, Backspin is another hierarchical dividing clustering algorithm, allowing to cluster both genes and cells (Zeisel et al., 2015). It uses the SPIN algorithm (Tsafrir et al., 2005) at each iteration to sort the expression matrix and then separates genes (rows) and cells (columns) into two groups by a specific splitting criterion. Alternatively, BISCUIT is a new iterative normalization and clustering procedure based on Dirichlet Process, which was designed to correct technical variation in scRNA-seq together with cell clustering (Prabhakaran et al., 2016).

### Graph approaches beyond clustering

Traditional clustering methods lack the function of inferring the inherent lineage between cells. Common approaches for cell lineage inferences require the creation of a graph or a tree, where single cells are represented as nodes and edges between the cells indicate their similarities. The lengths of the edges are computed from a similarity matrix based on a given metric. Before constructing the graph, a de-noising procedure is necessary. A useful de-noising procedure is to compute the *k*-Nearest-Neighbor graph (kNNG; Bendall et al., 2014; Levine et al., 2015; Xu and Su, 2015). Samples from the kNNG could then be compared using the geodesic distance, defined as the shortest path between two nodes (Bendall et al., 2014). Such an approach can remove “shortcuts” between irrelevant pairs of samples due to the curse of high dimensionality (Tenenbaum et al., 2000). Clustering analysis can then be performed on the graph using community detection algorithms (Fortunato, 2010). Xu and Su first used Euclidean distance to compute Shared Nearest-Neighbor (SNN) graph, then searched for quasi-cliques to obtain clusters of cells (Xu and Su, 2015). Quasi-cliques are communities of nodes, densely but not necessarily fully connected. Highly Connected Sub-graph (HPC) is another community detection algorithm that showed very similar performances as SNN (Hartuv and Shamir, 2000).

## Microevolution of single cells

### Inference without spatial and temporal information

scRNA-Seq data are also informative to reveal single-cell microevolution. Different algorithms have been specifically designed for scRNA-Seq to infer a pseudo temporal ordering of single cells. Moncole is the first scRNA-Seq bioinformatics tool to infer the temporal ordering of single cells (Trapnell et al., 2014). It first uses Independent Component Analysis (ICA) to reduce the dimension, then computes a Minimum Spanning Tree (MST) on the graph constructed by Euclidean distance between cell pairs. MST connects all nodes of a graph using edges with a minimal total weighting, based on the hypothesis that the longest path through the MST corresponds to the longest series of transcriptionally similar cells. Another similar method, Waterfall, uses PCA coupled with *k*-means to produce clusters, then connects the cluster centroids with MST (Shin et al., 2015). Similar to Waterfall, TSCAN is a new approach based on MST. Cells are first clustered using a model-based approach before constructing an MST, allowing the reduction of the tree space complexity (Ji and Ji, 2016).

Embeddr is a method that uses the correlation metric between cells to construct kNNG, then projects the samples into a low-dimensional embedding using Laplacian eigen maps. The pseudo time order is then fitted using the principal curves (Campbell et al., 2015). Embeddr aims to tackle the drawbacks of Monocle, where gene expression is modeled as a linear combination and the result is highly sensitive to outliers. This scheme is also used in the workflow of SLICER, a recent algorithm using Locally Linear Embedding (LLE) to project the dataset and to construct a kNNG among cells (Welch et al., 2016).

Since visualization is key in understanding reconstructed single-cell trajectories, better visualization algorithms are as important as methods to reconstruct the single-cell microevolution. *t*-SNE is a popular method to visualize single cells, as part of a more complex workflow (Jiang, L. et al., 2016; Petropoulos et al., 2016). Another approach derived from diffusion map was developed, allowing one to visualize a clear bifurcation event among the cells which may be missed by independent component analysis (ICA) or *t*-SNE (Haghverdi et al., 2015; Moignard et al., 2015).

### Modeling microevolution with spatial and temporal information

Cell subpopulations can also be characterized by different temporal and/or spatial gene expressions. Several approaches have been designed to exploit datasets with explicit temporal information. SCUBA is a method to detect bifurcation events using time course data (Marco et al., 2014). It assumes that the switch between cell states is a stochastic punctual process. To infer cellular hierarchy, it iteratively divides cells using *k*-means algorithm and uses a gap statistic to determine if a bifurcation event should occur. This process creates a binary tree, which can then be used to model gene expression dynamics (Marco et al., 2014). However, one drawback of SCUBA is that it requires data with temporal features. Free from such a requirement, Oscope is another method to infer oscillatory genes among single cells collected from a single tissue (Leng et al., 2015). It hypothesizes that these cells represent distinct states according to an oscillatory process. Oscope fits a two-dimensional sinusoidal function for each pair of genes, clusters gene pairs by frequency and reconstructs the order of the cells in a cyclic fashion. However, Oscope is unable to infer bifurcation events.

Other models also consider the spatial organization of cells in a tissue. Seurat is an approach that infers the spatial localization of single cells by integrating RNA-Seq with *in situ* RNA patterns (Satija et al., 2015). Seurat divides a cellular tissue into distinct spatial bins, linked by the expression of landmark genes per RNA *in-situ* hybridization. Within each bin, it builds a mixture model using expression values among correlated genes. The posterior probability is generated for each cell and assigned to a given bin. Another approach models the tissue as a 3D map and assumes that cells spatially close share common scRNA-Seq profiles (Pettit et al., 2014). This method uses a hidden markov random field to assign each bin of the map to a given cluster. Similar to Seurat, it takes the input of spatial gene expression measurement using whole mount *in situ* Hybridizations (WiSH) technology, a confocal microscopic approach that detects the presence of mRNA linked to a fluorescent probe.

## Challenges and future work

Compared to bulk-cell analysis, single-cell genomics has the advantage of exploring cellular processes with a more accurate resolution, but it is more vulnerable to disturbances. Besides perfecting the experimental protocols to deal with issues such as dropouts in gene expression and biases in amplification, deriving new analytical methods to reveal the complexity in scRNA-Seq data is just as challenging. In this review, we have listed the different bioinformatics algorithms dedicated to single-cell analysis. Although the initial few steps of workflow for scRNA-Seq analysis are similar to bulk-cell analysis (data pre-processing, batch removal, alignment, quality check, and normalization), the subsequent analyses are largely unique for single cells, such as subpopulations detection, and microevolution characterization (Figure 1). With the increasing popularity of single-cell assays and ever increasing number of computational methods developed, these methods need to be more accessible to research groups without bioinformatics expertise. Moreover, datasets where cell classes have already been previously charaterized should be identified as benchmark data, in order to accurately assess the performance of new bioinformatics methods.

Although this review focuses on scRNA-Seq analyses, with the rapid development of technologies, coupled DNA-based genomics data can be obtained from the same cell, in parallel with scRNA-Seq data (Han et al., 2014; Dey et al., 2015; Kim, K. T. et al., 2015; Macaulay et al., 2015). This will further increase the analytical challenges. Previous multi-omics bioinformatics tools applied to bulk samples could be leveraged. The use of graphs and tensor approaches that integrate heterogeneous features in bulk samples may be good starting points for multi-dimensional single cell data (Li et al., 2009; Levine et al., 2015; Katrib et al., 2016; Zhu et al., 2016). Efforts should also be made toward developing computational methods to make use of spatial information (possibly guided by imaging) in combination of scRNA-Seq (Pettit et al., 2014; Satija et al., 2015). Also most emphasis in scRNA-Seq by far has been made on protein coding genes, and the dynamics and roles of non-coding RNAs such as lncRNAs (Travers et al., 2015; Ching et al., 2016) and micro-RNAs are poorly explored. Finally, a large number of single-cells (*n* = 4645) in a single data set was reported recently (Tirosh et al., 2016), and the scRNA-Seq data volume is expected to continue growing exponentially. Foreseeably, this poses a large spectrum of challenges from developing more efficient aligners to better data storage and data sharing solutions.

## Author contributions

LG envisioned this project, OP, XZ, TC, and LG wrote the manuscript, all authors have read and agreed on the manuscript.

### Conflict of interest statement

The authors declare that the research was conducted in the absence of any commercial or financial relationships that could be construed as a potential conflict of interest.
